# Removal of Cr^6+^ ions from water by electrosorption on modified activated carbon fibre felt

**DOI:** 10.1098/rsos.180472

**Published:** 2018-09-26

**Authors:** Xinkun Zhao, Bingxin Jia, Qianqian Sun, Gaojie Jiao, Lili Liu, Diao She

**Affiliations:** 1 College of Resources and Environmental Sciences, Northwest A&F University, Yangling, Shaanxi 712100, People's Republic of China; 2 College of Forestry, Northwest A&F University, Yangling, Shaanxi 712100, People's Republic of China; 3 Institute of Soil and Water Conservation, Northwest A&F University, Yangling, Shaanxi 712100, People's Republic of China; 4 Beijing Zhongjing Tongchuang Energy Environment Technique Co., Ltd., Beijing 100049, People's Republic of China; 5 Research Center for Soil and Water Conservation and Eco-environmental Sciences, Chinese Academy of Sciences, Ministry of Education, Yangling, Shaanxi 712100, People's Republic of China

**Keywords:** activated carbon felt, chromium ion removal, nitric acid modification, electrosorption

## Abstract

Electrosorption is a novel desalination technique that has many advantages in the treatment of sewage. However, commercially available activated carbon electrodes for electrosorption commonly have low microporosity, poor moulding performance, and low adsorption and regeneration efficiency. Here, we evaluated a novel adsorbent material, activated carbon fibre felt (ACFF), for electrosorption of chromium ions (Cr^6+^) in sewage treatment. The ACFF was modified with 20% nitric acid and its modified structure was characterized. The modified ACFF was used as an adsorbing electrode to investigate its desalination effect by electrosorption. Results showed that compared with those of unmodified ACFF, the modified ACFF had more carbonyl and carboxyl groups and the specific surface area, average pore size and micropore volume of the modified ACFF also improved by 32.2%, 2.5% and 23.1%, respectively. The kinetics of Cr^6+^ adsorption conformed to the pseudo-second-order kinetic equation, and the adsorption isotherm conformed to the Langmuir model. In addition, the regeneration rate of the modified ACFF electrode was more than 94%. In conclusion, the modified ACFF exhibits excellent electrosorption and regeneration performance for Cr^6+^ removal from water and thus is of great value for promotion in sewage treatment.

## 1. Introduction

With the rapid development of the global economy, heavy metal contamination in water has become an increasingly prominent issue. Chromium (Cr) is a typical heavy metal contaminant that cannot be biologically transformed. This metal element often circulates through the food chain and accumulates in living organisms, causing a variety of disorders and diseases [1]. Therefore, the treatment of Cd contamination in water has become a hot topic of environmental research in recent years [2–7]. Available sewage treatment methods mainly include extraction, [2–5] ion exchange, [5–7] biological treatment [8] and electrosorption [9,10]. Among these methods, electrosorption is considered the most effective method for sewage treatment. The electrosorption technique applies electrical voltage to the electrode surface, which drives the charged ions to migrate towards the opposite electrode and adsorb onto it, thereby removing heavy metal ions [11–14].

Electrode material is a key factor in the electrosorption technique. The electrode for electrosorption is required to have high electrical conductivity, large specific surface area and stable electrochemical performance [15]. Johnson *et al*. [16] found that activated carbon has excellent electrical conductivity, large specific surface area and stable chemical performance. Satisfactory results were obtained in the removal of organic contaminants and heavy metal ions from water by electrosorption using activated carbon as the electrode material. However, activated carbon, when used as an electrode material for electrosorption, has a few disadvantages such as low microporosity, poor moulding performance, and low adsorption and regeneration efficiency [17–19].

Activated carbon fibre felt (ACFF) is a novel material, which is made of activated carbon fibre. ACFF is characterized by good electrical conductivity and chemical stability, with small and uniform pore size. This material has a high rate and velocity of adsorption for small molecules, while desorption readily occurs [20]. To date, ACFF has been frequently used as an adsorbent and extensively studied in air purification and gas adsorption. Peng *et al*. [21] showed that ACFF had a markedly high rate of gas adsorption, and that the purification efficiency of ACFF for formaldehyde, dust and other harmful substances was more than two times that of ordinary carbon fibre [22]. However, ACFF has rarely been studied as an adsorbent in sewage treatment.

During the preparation process, ash materials are often introduced into industrialized ACFF and plug the porous channels, seriously affecting the electrical conductivity and adsorption efficiency of the product. To solve this problem, ACFF needs to be pretreated before use for improving its adsorption performance [23]. Mei *et al*. [24,25] used ACFF modified with 20% nitric acid (HNO_3_) as an electrode and noted marked improvements in specific surface area, micropore number, and content of phenolic hydroxyl group and oxygen-containing functional groups; HNO_3_ modification also improved the electrical conductivity of ACFF and achieved the best modifying effect compared with other reagents. Furthermore, Mei *et al*. [24,25] showed that the modified ACFF achieved a remarkable effect for electrosorption of sulfonamides in water. However, the research about the electrosorption of inorganic contaminants such as heavy metal ions in water still was less.

In this study, we modified ACFF with 20% HNO_3_ and characterized its structure. The modified ACFF was used as an electrode for electrosorption of Cr^6+^ in water, in order to explore the effects of pH, voltage and plate spacing on the electrosorption performance of modified ACFF electrode. We analysed the electrosorption efficiency of the modified ACFF electrode and established its kinetic and isotherm models [26]. Finally, we analysed the regenerability of the modified ACFF electrode.

## 2. Material and methods

### 2.1. Modification of activated carbon fibre felt

The ACFF (Analytical reagent, Senyou Carbon Fiber Co., Ltd., Nantong, Jiangsu, China) was cut into 10 cm × 20 cm rectangled pieces. The materials were soaked in 1000 ml of deionized water for 2 h and then repeatedly rinsed with deionized water for 40 min. The washed materials were placed in a beaker containing 20% (W/V) HNO_3_ [27] and incubated on a thermostat oscillator for 2 h. Subsequently, the materials were washed with deionized water until the electrical conductivity of the washing liquid was lower than 10 µS cm^−1^. The materials were dried in an oven at 130°C for 10 h and then stored in a desiccator until use.

### 2.2. Preparation of electrosorption device

**Figure 1.**
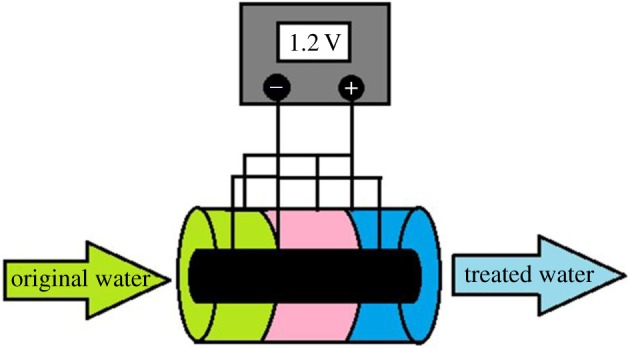
Diagram of electrosorption device used for removal of Cr^6+^ from water.

**Figure 2.**
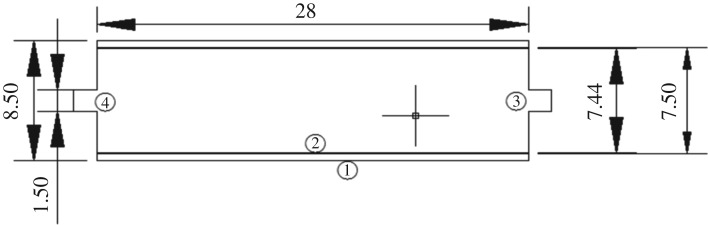
Plan of electrosorption device used for removal of Cr^6+^ from water (1. Stainless steel electrode, 2. Activated carbon fibre felt electrode, 3. Water inlet and 4. Water outlet).

The electrosorption device consisted of a direct-current power supply, a latex tube, a peristaltic pump, a liquid storage tank and a composite electrode (made of modified ACFF and stainless steel tube). The stainless steel was connected to the anode for the treatment of negatively charged contaminants and it was connected to the cathode for desorption and regeneration. During the treatment of Cr^6+^, Cr^6+^ ions were mainly present in the forms of Cr_2_O_7_^2–^, HCrO^4−^ and Cr_3_O_10_^2−^. The contaminants were negatively charged and the ACFF electrode was therefore connected to the cathode. The experimental device is shown in figures 1 and 2.

### 2.3. Electrosorption experiments

Single-factor experiments were conducted to analyse the effects of pH (2–10), initial concentration of Cr^6+^ (20–300 mg l^−1^), voltage (0.6–1.2 V), plate spacing (5–20 mm) on Cr^6+^ removal by electrosorption. One pair of electrodes were used in the electrosorption device for each experiment. Data were statistically analysed and multiple comparisons were performed using SPSS (Statistical Product and Service Solutions) (v. 19.0, IBM SPSS, Somers, NY, USA) [23].

### 2.4. Kinetic analysis of Cr^6+^ electrosorption

Electrosorption experiments were conducted using a 20 mg l^−1^ Cr^6+^ solution (pH 6) under the following conditions: voltage, 1.2 V; plate spacing, 5, 10, 15 and 20 mm; and flow rate, 0 ml h^−1^. The supernatant was collected at 2, 4, 6, 8, 10, 12, 14, 16, 18 and 20 h. The samples were filtered (0.45 µm Millipore filter) before the measurement of Cr^6+^ concentration in solution.

### 2.5. Thermodynamic analysis of Cr^6+^ electrosorption

Different concentrations (20, 60, 100, 200 and 300 mg l^−1^) of Cr^6+^ solution were prepared at pH 6. Electrosorption was conducted under the following conditions: temperature, 298 K; voltage, 1.2 V; flow rate, 0 ml h^−1^; and plate spacing, 5 mm. Twenty-four hours later, the supernatant was taken and filtered (0.45 µm pore filter). The Cr^6+^ concentration in solution was analysed as previously reported [28].

### 2.6. Cr^6+^ analysis in water

The concentration of Cr^6+^ ions in the experimental sewage was analysed by spectrophotometry using 2,2-diphenylcarbonic dihydrazide [26–28]. Standard Cr^6+^ solutions were prepared at the mass concentrations of 0, 0.5, 1, 2, 4, 8 and 16 mg l^−1^. The absorbance of the solutions at 540 nm wavelength was measured using a TU-1901 double-beam UV–Vis spectrophotometer (Beijing Ordinary General Analysis Instrument Co., Ltd., Beijing, China). The equation of the standard curve was obtained, *A* = 0.0082 × C[Cr^6^^+^] + 0.0007, where *A* is the absorbance at 540 nm wavelength and C_Cr_^6+^ is the concentration of Cr^6+^ (*R*^2^ = 0.9999).

### 2.7. Activated carbon fibre felt characterization

The surface morphology of ACFF samples was characterized using a FEI Quanta 200 scanning electron microscope (Carl Zeiss AG, Jena, Germany) under the following conditions: acceleration voltage, 3000 V; operating current, 10 µA; and working distance, 2.0–2.5 mm. N_2_ adsorption/desorption isotherms were obtained using an ASAP 2020 adsorption–desorption analyzer (Micromeritics instrument Corp., Atlanta, USA) at 77 K after samples were degassed at 105°C under 0.133 Pa for 24 h. The specific surface area, pore volume and pore size distribution of ACFF samples were calculated using the BET, t-plot and SF methods, respectively [18]. Fourier-transform infrared (FTIR) spectroscopy was performed using ACFF mixed with spectroscopically pure potassium bromide in a l : 100 (W/W) ratio. FTIR spectra were acquired using the tablet method [24].

### 2.8. Activated carbon fibre felt regeneration test

The electrosorption reactor was backwashed and regenerated using 20% HNO_3_ as the regenerant. The efficiency of backwashing and regeneration was compared between different electrodes (activated carbon, unmodified ACFF and modified ACFF) by experiments on a static oscillator. The modified ACFF electrode was allowed to reach saturated adsorption in 80 ml of 20 mg l^−1^ Cr^6+^ solution at the voltage of 1.2 V on the oscillator for 12 h and the adsorption capacity was then measured. Next, the electrodes at the ends of the plate were reversed [29] and regenerated in 20% HNO_3_ at 2 V for 2 h. After regeneration, the ACFF electrode was washed with deionized water followed by readsorption under the same conditions. The readsorption capacity was obtained and the regeneration rate of modified ACFF was calculated. The same approach was used to calculate the regeneration rate of activated carbon and unmodified ACFF electrodes. Each electrode was subjected to five adsorption-regeneration cycles. The regeneration rate was calculated as follows:

$$\text{regeneration rate}(\text{\%})=\frac{\text{readsorption capacity}}{\text{initial adsorption capacity}}\times 100$$

## 3. Results and discussion

### 3.1. Characteristics of activated carbon fibre felt

#### 3.1.1. Surface morphology

**Figure 3.**
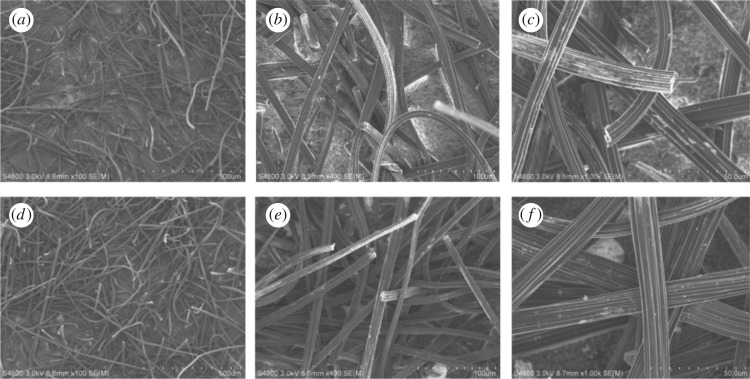
SEM images of activated carbon fibre felt before (*a–c*) and after modification with 20% HNO_3_ (*d–f*).

SEM images (figure 3*a–c*) show that before modification, the ACFF contained a large number of disorderly arranged, irregular fibre with normal shape and similar size, which also formed a small number of grooves to varying depths with discontinuities and irregular voids; the surface of the section was relatively flat and smooth. After being modified, the ACFF surface became more smooth and the longitudinal grooves were more prominent (figure 3*d–f*); this change in structure was conducive to increasing the specific surface area of the ACFF, thus providing more adsorption sites [30].

#### 3.1.2. Specific surface area and pore structure analysis

**Figure 4.**
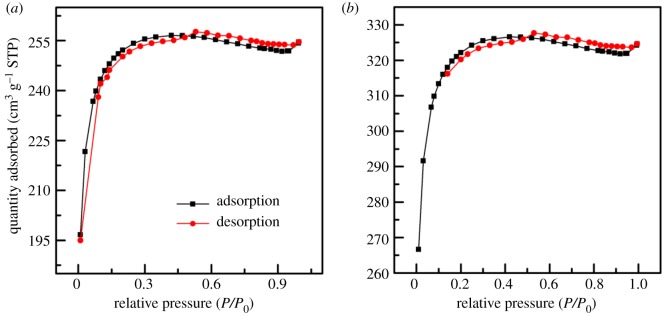
N_2_ adsorption–desorption isotherms of activated carbon fibre felt before (*a*) and after modification (*b*).

The N_2_ adsorption/desorption isotherms of unmodified and modified ACFF are shown in figure 4. It can be seen that microporous adsorption occurred in the low-pressure region; the curve rose rapidly with intensive single points. As the relative pressure *P*/*P*_0_ increased, the adsorption/desorption isotherms showed a retention loop. The adsorption capacity of ACFF increased rapidly only in a range of extremely low relative pressure and then gradually levelled off when a certain pressure was reached. Figure 4*b* shows that the modified ACFF maintained high adsorption–desorption capacity in the range of *P*/*P*_0_ < 1.0. According to the classification of adsorption isotherms proposed by the International Union of Pure and Applied Chemistry, the isotherms of modified ACFF were close to type IV and showed an H_4_ retention loop. This type of adsorption isotherm indicates that the modified ACFF contained a large number of micropore structure. All the isotherms showed a slight smearing, indicating the presence of mesopores in the ACFF [30].

The specific surface area, average pore size and micropore volume of modified ACFF improved to varying degrees, by 32.2%, 2.5% and 23.1% compared with those of unmodified ACFF (table 1). These improvements were also observed in the SEM images (figure 3). After the acid treatment, some tiny pores might have been connected, whereas ash and impurities were removed from the pores. Additionally, the activation at high temperature could create new pores and add the pore volume of some original pores, thus increasing the specific surface area of the ACFF.

**Table 1.** Specific surface area and pore distribution in activated carbon fibre felt (ACFF) before and after modification. Note: Different letters (‘a and b’) in the column indicate a highly significant difference before and after treatment (*p* < 0.01).

type	specific surface area (m^2^ g^−1^)	average pore size (nm)	micropore volume (cm^3^ g^−1^)
unmodified ACFF	835.26^b^	2.02^b^	0.26^b^
modified ACFF	1104.52^a^	2.07^a^	0.32^a^

#### 3.1.3. Fourier-transform infrared spectra

**Figure 5.**
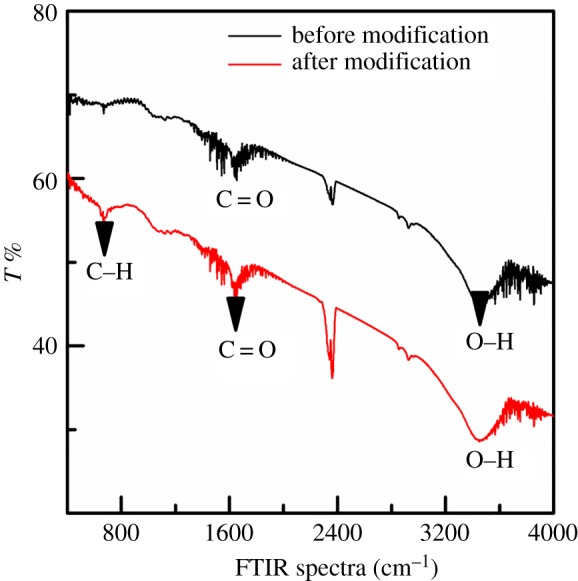
FTIR spectra of activated carbon fibre felt before and after modification.

The FTIR spectra of the ACFF before and after modification are shown in figure 5. Only three absorption peaks were found at 1760, 2360 and 3425 cm^−1^, respectively, in the spectra of the unmodified ACFF. The carbonyl absorption peak at 1700–1800 cm^−1^ was characteristic of infrared absorption peak of C = O, COOH or ester C = O; [31] the peak at 2300 cm^−1^ was attributed to CO_2_ [32]. After modification of ACFF, the peak intensity changed at 669, 1760 and 2360 cm^−1^, indicating an increase of sp^3^-hybridized carbon and hence an increase of defects on the felt surface during modification [31]. Moreover, the vibration peaks of oxygen-containing functional groups (1760 and 3425 cm^−1^) were significantly enhanced after felt modification, indicating an introduction of acidic oxygen-containing functional groups onto the surface, including carboxyl, hydroxyl and carbonyl groups. These groups, which are effective active sites for absorption, can greatly enhance the adsorption capacity of the adsorbent for Cr^6+^ [32].

### 3.2. Factors affecting Cr^6+^ electrosorption in water by modified activated carbon fibre felt

**Figure 6.**
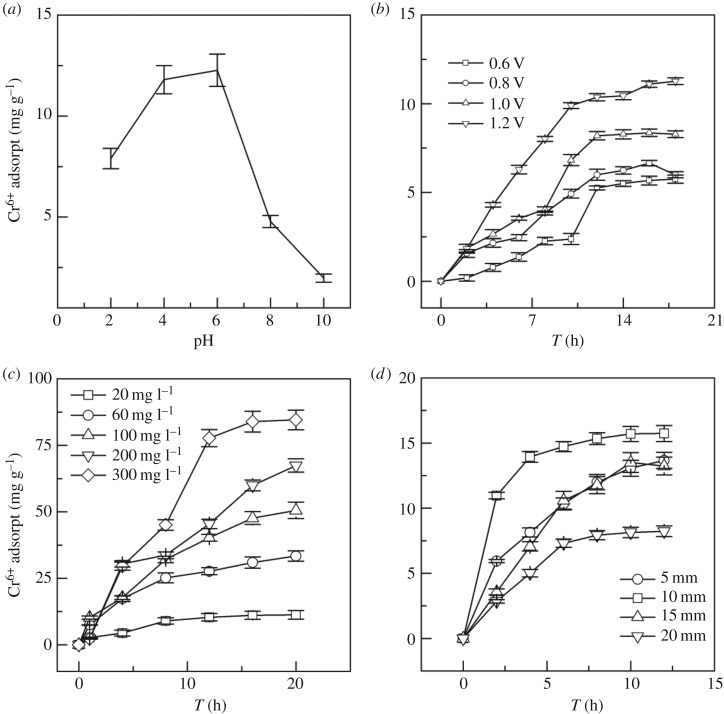
Effects of operating parameters on Cr^6+^ adsorption from water by electrosorption onto modified activated carbon fibre felt (*a*) pH; (*b*) voltage; (*c*) initial concentration of Cr^6+^; and (*d*) plate spacing.

The effects of different pH on Cr^6+^ electrosorption in water by the modified ACFF are shown in figure 6*a*. At pH < 4, the adsorption capacity of Cr^6+^ was relatively high but did not reach the maximum electrosorption capacity. At low pH, more positive charges were present on the surface of the modified ACFF, which resulted in a high electrostatic repulsion on the electrode surface. With increasing pH, the electrostatic repulsion on the electrode surface was reduced, so the Cr^6+^ adsorption capacity of the modified ACFF was increased [33]. The maximum unit adsorption capacity was achieved when the pH was raised to 6. Subsequently, the adsorption capacity of the modified ACFF for Cr^6+^ in water was drastically reduced due to an increase of pH. Meanwhile, the HO^−^ ions in solution competed with Cr_2_O_7_^2−^, HCrO_4_^−^ and Cr_3_O_10_^2−^ for adsorption sites under alkaline conditions, which also reduced the Cr^6+^ adsorption capacity. In practice, if wastewater has a neutral or slightly alkaline pH, it is necessary to adjust the pH to a slightly acidic level before heavy metal treatment using an electrosorption device.

The effects of voltage on the adsorption of Cr^6+^ (20 mg l^−1^) by the modified ACFF are shown in figure 6*b*. Within the range of voltage applied, the Cr^6+^ adsorption capacity of the modified ACFF markedly increased with increasing voltage. A higher voltage reinforced the electrosorption of Cr^6+^ onto the modified ACFF. When the voltage increased from 0.6 to 1.2 V, the Cr^6+^ adsorption capacity improved from 5.4 to 11.2 mg g^−1^ and the unit adsorption capacity improved by 107.4%, indicating a significant improvement in adsorption efficiency. An adsorption equilibrium was generally reached in all treatments after 12 h of adsorption. However, when the voltage was greater than 1.0 V, a small amount of bubbles occurred in water and this may be attributable to the weak electrolysis of water caused by the high voltage [34].

The electrosorption of Cr^6+^ in water at different initial concentrations by the modified ACFF is shown in figure 6*c*. As the initial concentration of Cr^6+^ increased, the adsorption capacity of the modified ACFF gradually increased. When the initial concentration of Cr^6+^ was 20 mg l^−1^, an adsorption equilibrium was reached at 10 h, with the adsorption capacity of 11.6 mg g^−1^. When the initial concentration of Cr^6+^ increased to 300 mg l^−1^, the Cr^6+^ adsorption capacity at an adsorption equilibrium improved to 84 mg g^−1^, because the chance of contact between Cr^6+^ and ACFF increased at high concentrations of Cr^6+^. Additionally, as the initial concentration of the contaminant increased, an increase occurred in the difference of the contaminant concentration between solution and ACFF surface. As a result, the tendency of contaminant migration towards the electrode was enhanced, thereby facilitating the contaminant adsorption and improving the adsorption capacity [34].

The effects of plate spacing on the adsorption of Cr^6+^ (300 mg l^−1^) by the modified ACFF are shown in figure 6*d*. The optimal adsorption effect was achieved with the plate spacing of 10 mm, because a smaller plate spacing allowed for the formation of a thicker electric double layer, which, in turn, shortened the migration distance of the charged ions to the electric double layer. However, an over-small spacing between the electrodes may hinder the flow of water, and possibly cause short circuit of the electrodes and increased energy consumption. Additionally, if the plate spacing is too small, there is no clear boundary between positive and negative ions and the freshwater area is very small, thus reducing the ion content in the outflow water. By contrast, if the plate spacing is too large, it takes a long time for the ions to reach and adsorb onto the electric double layer due to the long distance of ion diffusion between the electrodes and low turbulivity. Moreover, the electrode has limited effect on distant ions and cannot adsorb them onto its surface [35]. In summary, 10 mm was the optimal plate spacing for electrosorption of Cr^6+^ by the modified ACFF.

**Figure 7.**
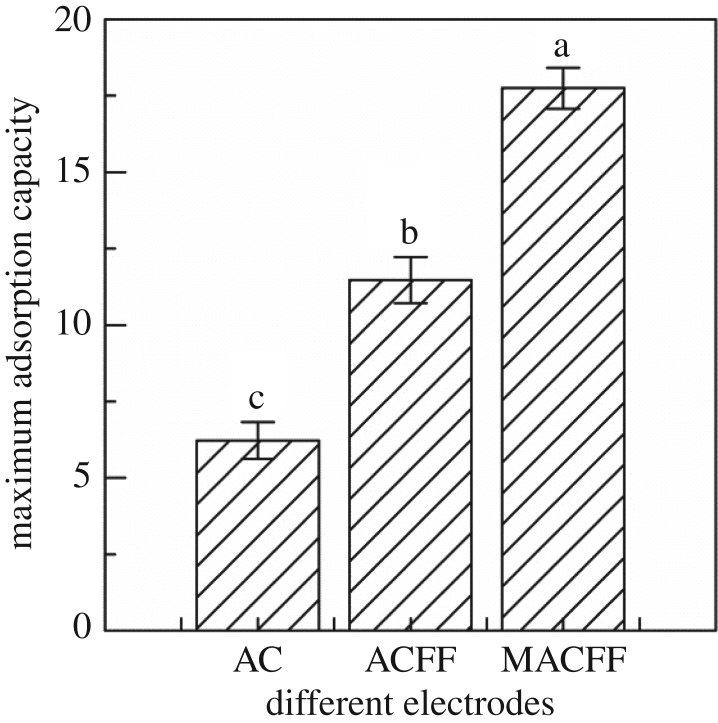
Maximum adsorption capacity of different electrodes (activated carbon, activated carbon fibre felt and modified activated carbon fibre felt) for Cr^6+^ (300 mg l^−1^) under optimal conditions.

The maximum adsorption capacity of different electrodes for Cr^6+^ (300 mg l^−1^) in water under the optimum conditions is shown in figure 7. The maximum adsorption capacity of activated carbon, unmodified ACFF and modified ACFF, as the adsorbing electrodes, was 6.22, 11.47 and 17.75 mg g^−1^, respectively. Compared with commercially available activated carbon, the ACFF showed a marked improvement in the maximum adsorption capacity either before or after modification, and the difference was highly significant (*p* < 0.01). ACFF has far larger specific surface area and pore number than activated carbon, while the adsorption sites increase accordingly in the former. Consequently, either modified or unmodified ACFF showed far higher adsorption capacity than activated carbon. Moreover, the modified ACFF showed a significant improvement in the maximum adsorption capacity for Cr^6+^ compared with the unmodified ACFF. This improvement could be attributable to the marked increases in specific surface area, micropore number and adsorption sites of the modified ACFF compared with the unmodified ACFF, in agreement with the conclusions from the above analysis (figures 3 and 5, and table 1).

### 3.3. Kinetics of Cr^6+^ electrosorption

**Figure 8.**
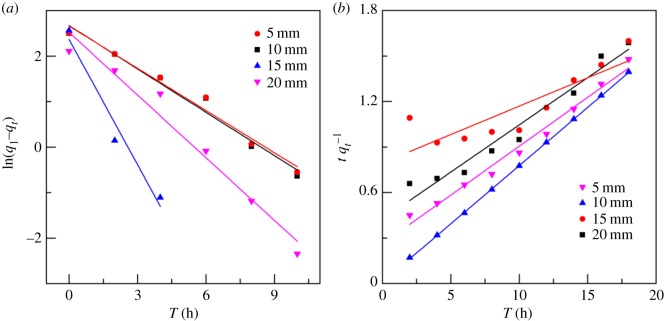
Kinetic equation fitting results of Cr^6+^ electrosorption onto modified activated carbon fibre felt at different plate spacings (*a*) pseudo-first-order kinetic model and (*b*) pseudo-second-order kinetic model.

The adsorption kinetic data were fitted with pseudo-first-order and pseudo-second-order kinetic models [15] (tables 2 and 3). The fitting results (figure 8) show that the pseudo-second-order kinetic model of Cr^6+^ electrosorption had higher *R*^2^ values (0.9256–0.9989) than the pseudo-first-order kinetic model (0.9348–0.9683). Compared with *q*_1_, *q*_2_ was also much closer to the measured *q*_e_. However, the figure and tables show that the first-order kinetic model could well describe the initial stage of Cr^6+^ electrosorption. As the electrosorption progressed, data were gradually derived from the fitted curve; the first-order kinetic equation was therefore not suitable to describe the entire process of Cr^6+^ electrosorption by the modified ACFF. As diffusion is an important factor limiting the first-order adsorption rate, our results suggest that the electrosorption of ions on the composite electrode was affected by resistance from liquid film diffusion. However, there was a large intercept on the fitted straight line, which indicates that liquid film diffusion was not the sole factor affecting the adsorption rate. Together, these results indicate that Cr^6+^ adsorption onto the modified ACFF is a complex chemical adsorption process. As the second-order kinetic model covered all the adsorption processes, this model could more fully reflect the dynamic mechanism of ion adsorption onto the composite electrode [36].

**Table 2.** Fitting parameters of the pseudo-first-order kinetic equation for Cr^6+^ electrosorption onto modified activated carbon fibre felt.

plate spacing (mm)	*q*_e_ (mg g^−1^)	*q*_l_ (mg g^−1^)	*K*_1_ (min^−1^)	*R*^2^
20	8.33	11.372	−0.439	0.9683
15	5.65	15.37	−0.2258	0.9348
10	13.91	11.459	−0.9570	0.9366
5	13.17	13.31	−0.3190	0.9645

**Table 3.** Fitting parameters of the pseudo-second-order kinetic equation for Cr^6+^ electrosorption onto modified activated carbon fibre felt.

plate spacing (mm)	*q*_e_ (mg g^−1^)	*q*_2_ (mg g^−1^)	*K*_2_ (g (mg min)^−1^)	*R*^2^
20	8.47	15.05	0.057	0.9732
15	5.75	6.97	0.1691	0.9256
10	12.54	12.97	0.0747	0.9989
5	12.46	14.92	0.0674	0.9874

### 3.4. Thermodynamics of Cr^6+^ electrosorption

**Figure 9.**
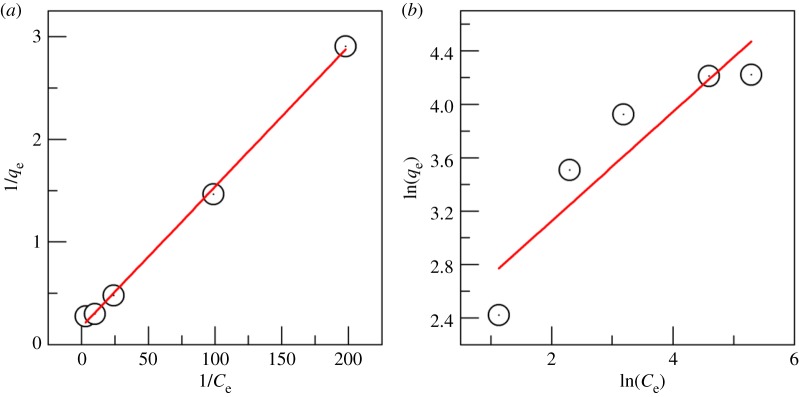
Linear fitting of isothermal models for Cr^6+^ electrosorption onto modified activated carbon fibre felt: (*a*) Langmuir model and (*b*) Freundlich model.

The adsorption isotherm data of Cr^6+^ electrosorption by the modified ACFF were fitted with the Langumir and Freundlich models [37]. Figure 9 and table 4 show that the Langumir model could better describe the electrosorption behaviour of the modified ACFF for Cr^6+^. In the Langumir model, RL = 0.089 indicates preferential adsorption; in the Freundlich model, *n* = 2.39 also indicates preferential adsorption, and the same conclusion can be drawn. –Cr^6+^ adsorption on the modified ACFF was preferential adsorption under the experimental conditions. The Freundlich model was slightly inferior to the Langumir model for fitting the adsorption isotherm data, suggesting that Cr^6+^ adsorption tended to form a monomolecular layer on the modified ACFF.

**Table 4.** Freundlich and Langmuir models of Cr^6+^ electrosorption onto modified activated carbon fibre felt.

isothermal model	fitting equation	correlation coefficient		
Freundlich	*y* = 0.4179*x* + 2.7547	*n* *=* *2.39*	*k*_f_ *=* *15.71*	*R*^2^ = 0.79432
Langmuir	*y* = 0.0142*x* + 0.0132	*q_m_* *=* *75*	*k*_L_ *=* *1.06*	*R*^2^ = 0.9968

### 3.5. The intraparticle diffusion model

**Figure 10.**
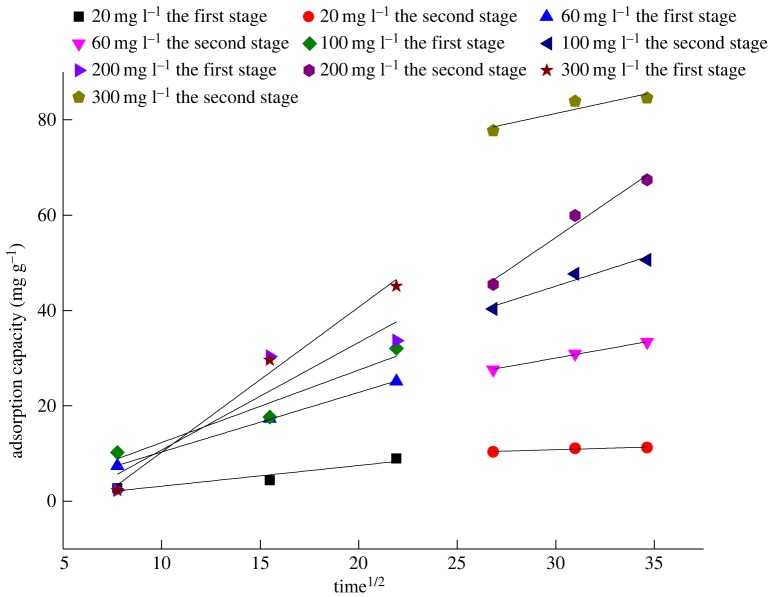
Model of internal diffusion of Cr^6+^ electrostatic sorption in ACFF.

As shown in figure 10 and table 5, the equation of the intraparticle diffusion model did not pass the origin. The result indicated that there are two key processes during the adsorption, namely membrane diffusion and internal diffusion. At the beginning of adsorption, it was the surface diffusion stage. There was a relatively high adsorption rate and large adsorption power at this stage, which were mainly due to that fact that the surface adsorption site of the activated carbon felt is sufficient and there was a relatively high Cr^6+^ content in the solution. Therefore, the main influencing factor of the intraparticle diffusion step is the adsorption power. With the increase of Cr^6+^ content in the solution, the adsorption power observably increased and the slope of the equation also became larger (figure 10 and table 5). After the adsorption time reached 480 min, it would be into the internal diffusion stage. The adsorption power was weaker due to the relatively fewer adsorption sites and low Cr^6+^ content. This stage depended on the membrane diffusion and internal diffusion. At the final stages of adsorption, it would reach equilibrium owing to the almost saturated adsorption sites.

**Table 5.** Model of internal diffusion of Cr^6+^ electrostatic sorption in ACFF.

the initial concentration of Cr^6+^ (mg l^−1^)	Ki1	*R*^2^	Ki2	*R*^2^
20	0.4342	0.8092	0.1164	0.8026
60	1.2506	0.9997	0.7389	0.9958
100	1.5206	0.8899	1.3277	0.911
200	2.2564	0.7332	2.8219	0.9576
300	3.0429	0.9788	0.8898	0.7009

### 3.6. Backwashing and regeneration of electrosorption reactor

**Figure 11.**
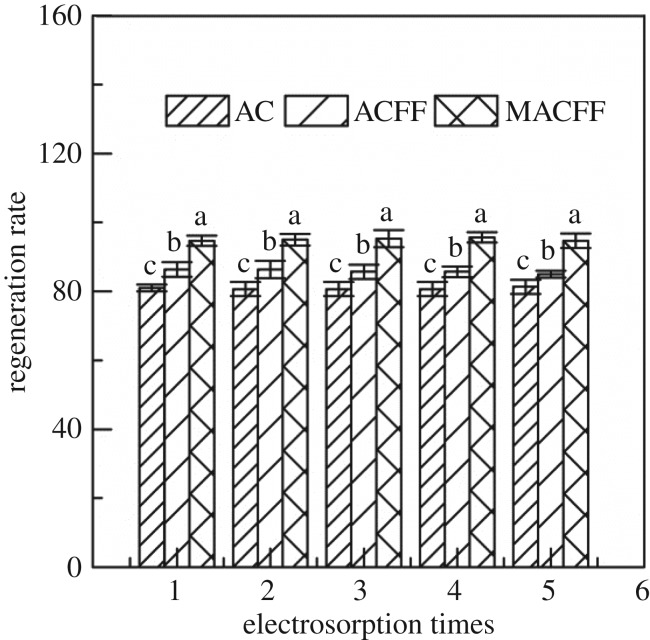
Regeneration rate of different electrodes used for Cr^6+^ electrosorption. Data are means ± s.d. (*n* = 3). Different letters above the column indicate significant difference at *p* < 0.01.

The regeneration rate of different adsorbing electrodes is shown in figure 11. After five cycles of backwashing and regeneration, the regeneration rates of activated carbon, unmodified ACFF and modified ACFF were maintained at approximately 81%, 87% and 94%, respectively. The modified ACFF showed a markedly higher regeneration rate than the activated carbon and unmodified ACFF, and the difference was highly significant. The electrode sheet remained intact in the process of backwashing and regeneration. No scaling or passivation was observed. The regeneration rate did not significantly change with increasing times of backwashing and regeneration, indicating that the modified ACFF had a desalination cycle and a long service life [38].

## 4. Conclusion

The ACFF treated with 20% HNO_3_ showed improvements in specific surface area, average pore size and micropore volume. The modified ACFF also had an increased number of carbonyl and carboxyl groups, which provided more active adsorption sites. The optimal working conditions for electrosorption Cr^6+^ by the modified ACFF electrode were: voltage, 1.2 V; pH, 6; and plate spacing, 10 mm. The kinetics of Cr^6+^ electrosorption on the modified ACFF electrode conformed to the pseudo-second kinetic equation, and the electrosorption isotherm conformed to the Langmuir model. These findings provide evidence for further exploring the inherent mechanism of desalination by electrosorption. The regeneration rate of the modified ACFF was approximately 94% after multiple adsorption-regeneration cycles by polarity reversal, with 20% HNO_3_ solution as the regenerant. The ACFF electrode showed excellent regenerability and could greatly reduce the application costs of the desalination technique by electrosorption, which provided theoretical support for large-scale application of this technique.

## Data accessibility

A complete dataset of measurements is available from Dryad: https://doi.org/10.5061/dryad.m0c5820.

## References

[1] Qian Z, Qi P, Wang G 2005 Main revisions on Chinese pharmacopoeia (2005 edition), volume I Beijing, China: Drug Stanoaros of China.

[2] Vieno NM, Tuhkanen T, Kronberg L 2006 Analysis of neutral and basic pharmaceuticals in sewage treatment plants and in recipient rivers using solid phase extraction and liquid chromatography-tandem mass spectrometry detection. J. Chromatogr. A 1134, 101–111. (10.1016/j.chroma.2006.08.077) 16996072

[3] Okuda T, Yamashita N, Tanaka H, Matsukawa H, Tanabe K 2009 Development of extraction method of pharmaceuticals and their occurrences found in Japanese wastewater treatment plants. Environ. Int. 35, 815–820. (10.1016/j.envint.2009.01.006) 19201472

[4] Bicchi C, Schilirò T, Pignata C, Fea E, Cordero C, Canale F 2009 Analysis of environmental endocrine disrupting chemicals using the e-screen method and stir bar sorptive extraction in wastewater treatment plant effluents. Sci. Total Environ. 407, 1842 (10.1016/j.scitotenv.2008.11.039) 19101021

[5] Limcaco CA 2011 System and method for biological wastewater treatment and for using the byproduct thereof. US, US 7981292 B2.

[6] Lin SH, Chen ML 1997 Textile wastewater treatment by enhanced electrochemical method and ion exchange. Environ. Technol. Lett. 18, 739–746. (10.1080/09593331808616592)

[7] Jilai LU, Cao L, Zhou H, Yong TU, Liu W 2013 Nickel plating rinse wastewater treatment by ion-exchange method. Industrial Safety & Environmental Protection 39, 13–15.

[8] Dionisi D, Rasheed AA, Majumder A 2016 A new method to calculate the periodic steady state of sequencing batch reactors for biological wastewater treatment: model development and applications. J. Environ. Chem. Eng. 4, 3665–3680. (10.1016/j.jece.2016.07.032)

[9] Figueroa FL, Jerez CG, Korbee N 2013 Use of in vivo chlorophyll fluorescence to estimate photosynthetic activity and biomass productivity in microalgae grown in different culture systems. Latin Am. J. Aquat. Res. 41, 801–819. (doi:103856/vol41-issue5-fulltext-1)

[10] Hughes JD 2013 Responses to natural disasters in the Greek and roman world, pp. 111–137. Dordrecht: Springer.

[11] Antov Y, Barbul A, Mantsur H, Korenstein R 2005 Electroendocytosis: exposure of cells to pulsed low electric fields enhances adsorption and uptake of macromolecules. Biophys. J. 88, 2206 (10.1529/biophysj.104.051268) 15556977 PMC1305271

[12] Tanahashi I, Yoshida A, Nishino A 1991 Characterization of activated carbon fiber cloths for electric double-layer capacitors by adsorption method. Carbon 29, 1033–1037. (10.1016/0008-6223(91)90183)

[13] Kreuzer HJ, Wang LC, Lang ND 1992 Self-consistent calculation of atomic adsorption on metals in high electric fields. Phys. Rev. B: Condens. Matter 45, 12 050–12 055. (10.1103/PhysRevB.45.12050) 10001224

[14] Petkovska M, Antov-Bozalo D, Markovic A, Sullivan P 2007 Multiphysics modeling of electric-swing adsorption system with in-vessel condensation. Adsorption-J. Int. Adsorption Soc. 13, 357–372. (10.1007/s10450-007-9028-2)

[15] Selvakumaran J, Hughes MP, Keddie JL, Ewins DJ 2002 Assessing biocompatibility of materials for implantable microelectrodes using cytotoxicity and protein adsorption studies. In Microtechnologies in Medicine & Biology, International Ieee-Emb Special Topic Conference on, pp. 261–264. IEEE.

[16] Bakshi BR 2011 The path to a sustainable chemical industry: progress and problems. Curr. Opin. Chem. Eng. 1, 64–68. (10.1016/j.coche.2011.07.004)

[17] Zhang Q, Zhang ZH, Wang L, Zhang ZL, Guo XF 2012 Effect of ACF properties on the electric adsorption performance of the ACF electrode. Appl. Mech. Mater. 209–211, 1990–1994. (10.4028/www.scientific.net/AMM.209-211.1990)

[18] Shiraishi S, Nakajima T, Kurihara H, Ozaki JI, Oya A 2010 Influence of organics adsorption on electric double layer capacitance for activated carbon electrode. Tanso 2004, 255–257. (10.7209/tanso.2004.255)

[19] Hasegawa G 2013 Monolithic electrode for electric double-layer capacitors based on macro/meso/microporous S-containing activated carbon with high surface area. J. Mater. Chem. 21, 2060–2063.

[20] Luo L, Ramirez D, Rood MJ, Grevillot G, Hay KJ, Thurston DL 2006 Adsorption and electrothermal desorption of organic vapors using activated carbon adsorbents with novel morphologies. Carbon 44, 2715–2723. (10.1016/j.carbon.2006.04.007)

[21] Fathy NA, El-Wakeel ST, El-Latif RRA 2015 Biosorption and desorption studies on chromium(vi) by novel biosorbents of raw rutin and rutin resin. J. Environ. Chem. Eng. 3, 1137–1145. (10.1016/j.jece.2015.04.011)

[22] Daifullah AAM, Girgis BS 2003 Impact of surface characteristics of activated carbon on adsorption of BTEX. Colloids Surf. A Physicochem. Eng. Aspects 214, 181–193. (10.1016/S0927-7757(02)00392-8)

[23] García-Araya JF, Beltrán FJ, Álvarez P, Masa FJ 2003 Activated carbon adsorption of some phenolic compounds present in agroindustrial wastewater. Adsorption-J. Int. Adsorption Soc. 9, 107–115. (10.1023/A:1024228708675)

[24] Zhang C X, Zhang R, Xing BL, Cheng G, Xie YB, Qiao WM 2010 Effect of pore structure on the electrochemical performance of coal-based activated carbons in non-aqueous electrolyte. New Carbon Mater. 25, 129–133. (10.1016/S1872-5805(09)60020-2)

[25] Matsui Y, Aizawa T, Suzuki M, Kawase Y 2007 Removal of geosmin and algae by ceramic membrane filtration with super-powdered activated carbon adsorption pretreatment. Water Sci. Technol. Water Supply 7, 43–51. (10.2166/ws.2007.080)

[26] Kuchta B, Firlej L, Marzec M, Boulet P 2008 Modeling of adsorption in pores with strongly heterogeneous walls: parametric lattice-site wall model. Adsorption-J. Int. Adsorption Soc. 14, 201–205. (10.1007/s10450-007-9078-5)

[27] Matsuda H, Bernardo EC, Fukuta T, Fujita T, Matsubara T, Kojima Y 2005 Effect of ultraviolet irradiation pretreatment on the removal of saccharin by activated carbon adsorption. Asian Pacific Confederation of Chemical Engineering Congress Program and abstracts 2004, 100–1000. (doi:10.11491/apcche.2004.0.1000.0)

[28] Pei CJ, Yao GG, Zhou FL, Zhang WC 2009 Determination of chromium (VI) in water by ultraviolet spectrophotometry. Hubei Agric. Sci. 48, 968–970.

[29] Jacobs DA, Wu Y, Shen H, Barugkin C, Beck FJ, White TP 2017 Hysteresis phenomena in perovskite solar cells: the many and varied effects of ionic accumulation. Phys. Chem. Chem. Phys. 19, 3094–3103. (10.1039/C6CP06989D) 28079207

[30] Blok WJ G D, Mcgaugh SS 1997 The dark and visible matter content of low surface brightness disc galaxies. Monthly Notices R. Astron. Soc. 290, 533–552. (10.1093/mnras/290.3.533)

[31] Zhang ZY, Xu XC 2015 Nondestructive covalent functionalization of carbon nanotubes by selective oxidation of the original defects with K2FeO4. Appl. Surf. Sci. 346, 520–527. (10.1016/j.apsusc.2015.04.026)

[32] Saharan P, Chaudhary GR, Mehta SK, Umar A 2014 Removal of water contaminants by iron oxide nanomaterials. J. Nanosci. Nanotechnol. 14, 627–643. (10.1166/jnn.2014.9053) 24730287

[33] Yang ST, Zhao DL, Zhang H, Lu SS, Chen L, Yu XJ 2010 Impact of environmental conditions on the sorption behavior of Pb(ii) in Na-bentonite suspensions. J. Hazard. Mater. 183, 632–640. (10.1016/j.jhazmat.2010.07.072) 20728269

[34] Thirumavalavan M, Lai YL, Lin LC, Lee JF 2010 Cellulose-based native and surface modified fruit peels for the adsorption of heavy metal ions from aqueous solution: Langmuir adsorption isotherms. J. Chem. Eng. Data 55, 1186–1192. (10.1021/je900585t)

[35] Takeda K, Uchihashi T, Watanabe H, Ishida T, Igarashi K, Nakamura N 2015 Real-time dynamic adsorption processes of cytochrome c on an electrode observed through electrochemical high-speed atomic force microscopy. PLoS ONE 10, e0116685 (10.1371/journal.pone.0116685) 25671430 PMC4324961

[36] Yoosefian M, Ahmadzadeh S, Aghasi M, Dolatabadi M 2017 Optimization of electrocoagulation process for efficient removal of ciprofloxacin antibiotic using iron electrode; kinetic and isotherm studies of adsorption. J. Mol. Liq. 225, 544–553. (10.1016/j.molliq.2016.11.093)

[37] Shukla A, Sahoo S, Moharir AS 2017 Non-isothermal multi-cell model for pressure swing adsorption process. Int. J. Hydrog. Energ. 23, 515–534. (10.1016/j.ijhydene.2016.11.200)

[38] Myint MTZ, Dutta J 2012 Fabrication of zinc oxide nanorods modified activated carbon cloth electrode for desalination of brackish water using capacitive deionization approach. Desalination 305, 24–30. (10.1016/j.desal.2012.08.010)

